# Pancreatic Steatosis as a Risk Phenotype for Pancreatic Ductal Adenocarcinoma: A Narrative Review

**DOI:** 10.3390/medicina62040729

**Published:** 2026-04-10

**Authors:** Roberto Cammarata, Vincenzo La Vaccara, Lucrezia Bani, Federica Giordano, Pierpaolo Castagliuolo, Maria Vittoria Ristori, Sara Elsa Aita, Silvia Angeletti, Roberto Coppola, Damiano Caputo

**Affiliations:** 1 Operative Research Unit of General Surgery, Fondazione Policlinico Universitario Campus Bio-Medico, 00128 Rome, Italy; 2 Faculty of Medicine and Surgery, Sapienza University of Rome, 00185 Rome, Italy; 3 Department of Surgery, Università Campus Bio-Medico di Roma, 00128 Rome, Italy; 4 Research Unit of Clinical Laboratory Science, Department of Medicine and Surgery, Università Campus Bio-Medico di Roma, Via Alvaro del Portillo, 21, 00128 Rome, Italy; 5 Operative Research Unit of Laparoscopic and Minimally Invasive Surgery, Fondazione Policlinico Universitario Campus Bio-Medico, Via Alvaro del Portillo, 200, 00128 Rome, Italy; 6 Operative Research Unit of Laboratory, Fondazione Policlinico Universitario Campus Bio-Medico, Via Alvaro del Portillo, 200, 00128 Rome, Italy; 7 Research Unit of General Surgery, Università Campus Bio-Medico di Roma, 00128 Rome, Italy

**Keywords:** pancreatic ductal adenocarcinoma, pancreatic cancer, PDAC, pancreatic steatosis, fatty pancreas, pancreatic fat, intrapancreatic fat, IPFD

## Abstract

*Background and Objectives*: Pancreatic ductal adenocarcinoma (PDAC) is one of the leading causes of cancer-related mortality, largely due to late-stage diagnosis and the absence of effective population-based screening. Intrapancreatic fat deposition (IPFD) has emerged as a potential risk phenotype. This narrative review critically appraises the clinical, metabolic, epidemiologic, and mechanistic evidence linking IPFD to PDAC and discusses its implications for risk stratification and prevention. *Materials and Methods*: A structured literature search was conducted in PubMed/MEDLINE and Scopus for studies published between 2007 and 2025 using predefined terms related to pancreatic steatosis and pancreatic cancer. After duplicate removal and screening according to predefined inclusion and exclusion criteria, 42 articles were included. Evidence was synthesized focusing on epidemiologic associations, mechanistic pathways, and imaging-based quantification methods. *Results*: A strong association between IPFD and PDAC was found. Although definitive causality remains unproven, some studies support temporal correlation between IPFD and PDAC, suggesting that IPFD precedes PDAC. A possible pathophysiological explanation to this correlation has been advanced in experimental models indicating IPFD as a pro-inflammatory factor cooperating with oncogenic KRAS to facilitate neoplastic progression. Finally, variability in IPFD definitions and heterogeneity in imaging assessment limit interpretability. *Conclusions*: Current evidence links IPFD to PDAC risk, suggesting a strong suspicion that pancreatic steatosis may represent an independent risk factor for PDAC. Still robust causal inference remains unproven. Well-designed prospective studies, standardized imaging protocols, and mechanistic investigations are required to clarify causality and determine whether pancreatic steatosis can be incorporated into risk-based screening and preventive strategies.

## 1. Introduction

Pancreatic ductal adenocarcinoma (PDAC) is one of the leading causes of cancer-related mortality worldwide, with a five-year survival rate of approximately 13%, and it is projected to become the second leading cause of cancer death by 2030 in the United States [1,2]. This lethality is mainly driven by late-stage diagnosis, as most patients are diagnosed with locally advanced (30–35%) or metastatic disease (50–55%), when curative treatment is no longer feasible [3,4]. In the absence of population-based screening programs and validated markers for risk stratification in asymptomatic individuals, identifying detectable and potentially modifiable conditions that precede malignant transformation represents a key opportunity to shift PDAC management from late-stage treatment toward earlier interception.

In this context, pancreatic steatosis—also referred to as intra-pancreatic fat deposition or fatty pancreas—has emerged as a potential risk phenotype of growing interest. Once regarded as an incidental imaging finding, IPFD is increasingly recognized as a biologically relevant condition with a pooled prevalence of approximately 21% and associations with adverse metabolic features, diabetes, acute pancreatitis, and pancreatic cancer [1,5]. Accumulating evidence from case–control studies, retrospective cohorts, systematic reviews, and meta-analyses suggests that pancreatic steatosis may represent an independent risk factor for PDAC rather than a simple consequence of obesity and metabolic syndrome [6,7,8,9]. Moreover, pancreatic steatosis has been proposed both as a potential early and modifiable pre-neoplastic niche preceding pancreatic ductal adenocarcinoma development; however, these interpretations remain largely hypothetical and require validation through robust prospective, predictive cohort studies and dedicated mechanistic investigations [10,11].

This narrative review provides an integrated synthesis of the evidence linking intrapancreatic fat deposition to PDAC, tracing the progression from epidemiological association to causal inference, critically appraising imaging-based quantification methods, incorporating compartment-specific fat distribution data, and discussing the implications for risk stratification and prevention.

## 2. Methods

To inform this structured narrative review, a literature search was conducted using two electronic databases, PubMed/MEDLINE and Scopus. The following search terms were applied: (“pancreatic steatosis” OR “fatty pancreas” OR “pancreatic fat” OR “intrapancreatic fat” OR “pancreatic lipomatosis” OR “non-alcoholic fatty pancreas disease” OR “NAFPD”) AND (“pancreatic cancer” OR “pancreatic ductal adenocarcinoma” OR “PDAC”) AND (“clinical” OR “metabolic” OR “inflammation” OR “insulin resistance” OR “metabolic syndrome” OR “new-onset diabetes” OR “early detection” OR “prevention”).

This search yielded 71 articles in PubMed and 122 articles in Scopus, covering publications from 2007 to 2025. After removal of duplicates, 133 unique records were identified.

Eligible studies included peer-reviewed clinical, imaging, histological, or experimental investigations, as well as relevant reviews, examining pancreatic steatosis (including fatty pancreas and related terms) in relation to pancreatic ductal adenocarcinoma, pancreatic neoplasms, or oncogenesis-related biological processes. Case reports; purely descriptive or non-oncological studies; editorials, letters, conference abstracts, preprints, non-English publications, generic reviews without specific relevance to pancreatic cancer, duplicate publications, secondary analyses without novel data, and studies superseded by higher-quality investigations were excluded.

**Figure 1:**
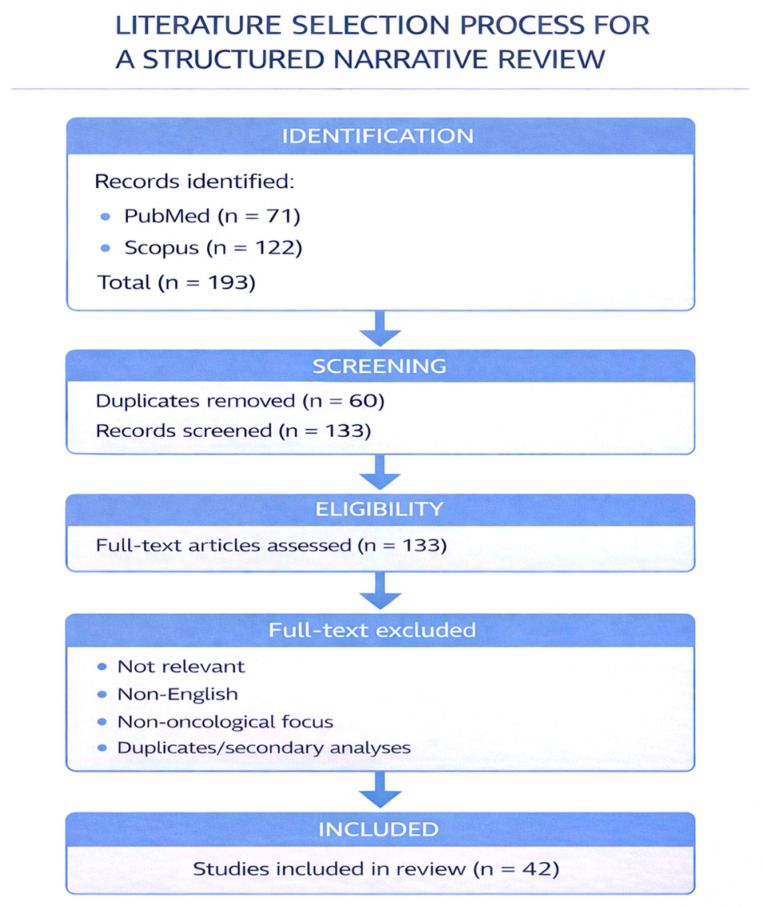
PRIMA-style flow diagram.

Following this screening process, 42 articles were included in the final synthesis. The main reasons for exclusion were lack of relevance to the specific research question, non-English language, and overly generic focus, as shown in Figure 1.

The main studies with study design, cohort size, and key findings were summarized in Table 1.

## 3. Background

Pancreatic steatosis is the accumulation of fat within the pancreatic parenchyma. It was first described in 1933 in a post-mortem study by Ogilvie, who reported greater fat deposition in the pancreas of obese and older individuals, along with associations between marked fatty infiltration and metabolic and vascular comorbidities, including type 2 diabetes mellitus and severe atherosclerosis [27]. However, these early observations, although historically important, were derived from descriptive autopsy studies and therefore could neither establish causality nor clarify whether pancreatic fat represented a driver of disease or merely a marker of systemic metabolic dysfunction.

One of the first human studies to move beyond purely descriptive evidence was published in 2009 by Mathur et al., who reported that increased intrapancreatic adipocyte content—assessed histologically in resected specimens—was associated with lymph node positivity and reduced survival in patients with pancreatic ductal adenocarcinoma, independently of body mass index. Although limited by its retrospective design and post-diagnostic setting, this study introduced the hypothesis that pancreatic steatosis might influence tumor aggressiveness and dissemination [7].

Building on these observations, Smits and van Geenen in 2011 critically summarized the emerging literature on pancreatic steatosis, framing it as a biologically plausible but still unproven disease modifier and underscoring the need for longitudinal and mechanistic studies. They were among the first to conceptualize pancreatic steatosis as a condition potentially implicated in metabolic disease, pancreatitis, and pancreatic cancer, thereby moving the field beyond the notion of a purely incidental or bystander phenomenon [28]. These uncertainties were subsequently reiterated in comprehensive narrative reviews, which consistently underscored the heterogeneity in definitions, diagnostic approaches, and reported clinical associations of pancreatic steatosis, and called for longitudinal studies to clarify its biological and clinical relevance [29,30,31,32,33].

More recently, Petrov published a Personal View in The Lancet Gastroenterology & Hepatology (2023), proposing a conceptual reappraisal of intrapancreatic fat deposition as a unifying pathogenic substrate across pancreatic diseases, including pancreatic cancer, formalized as the PANDORA (pancreatic diseases originating from intrapancreatic fat) hypothesis [34].

## 4. Pathophysiology

### 4.1. Mechanisms of Pancreatic Fat Accumulation: Fatty Infiltration Versus Fatty Replacement

Pancreatic steatosis can develop through two main pathways: fatty infiltration and fatty replacement. Fatty infiltration refers to the ectopic accumulation of adipose tissue within the pancreatic parenchyma and is typically associated with metabolic syndrome, visceral obesity, insulin resistance, and a sedentary lifestyle. Fatty replacement, instead, results from pancreatic injury and involves the loss of acinar cells with their replacement by adipocytes, as observed in conditions such as chronic pancreatitis, alcohol-related damage, viral infections, malnutrition, and use of corticosteroids or chemotherapeutic therapies [1,35,36].

### 4.2. Anatomical Distribution of Pancreatic Fat: Intralobular and Extralobular Compartments

Recent mechanistic analyses of human pancreatic tissue indicate that pancreatic steatosis is biologically heterogeneous. Integrated lipidomic, transcriptomic, and histopathological data show that intralobular fat (ILF) is primarily associated with obesity-related acinar stress and inflammation, whereas extralobular fat (ELF) correlates more closely with PanIN burden and fibrotic remodeling, suggesting involvement at different stages of pancreatic carcinogenesis. These compartment-specific effects may be obscured when pancreatic steatosis is assessed as a single entity in imaging and epidemiological studies. However, the study is limited by its cross-sectional design, lack of normal pancreatic controls, small sample size, incomplete lipid characterization, and the use of separate cohorts for different omics analyses, limiting causal and temporal interpretation [22].

### 4.3. Pancreatic Steatosis as a Permissive Microenvironment for Carcinogenesis

In Onohuean H. et al., pancreatic steatosis was proposed to promote pancreatic carcinogenesis through the convergence of lipotoxic stress, chronic low-grade inflammation, stromal remodeling, and immune dysregulation, thereby creating a permissive metabolic and inflammatory microenvironment that lowers the biological threshold for KRAS-driven neoplastic transformation rather than directly inducing oncogenic mutations [10].

In line with this paradigm, mechanistic insights from obesity-driven experimental models suggest that intrapancreatic fat may act as a local paracrine source of adipokines, free fatty acids, and inflammatory mediators that cooperate with oncogenic KRAS to facilitate early pancreatic carcinogenesis, while remaining insufficient on their own to initiate malignant transformation [37].

Within this context, preclinical evidence suggests that the steatotic pancreas is normally buffered by endogenous metabolic defense mechanisms—such as fibroblast growth factor 21 (FGF21)—and that oncogenic KRAS-mediated suppression of these protective pathways may favor inflammation-associated fibro-fatty remodeling and accelerate neoplastic progression, thereby raising the possibility that restoration of such metabolic defenses could represent a future therapeutic strategy [38].

From a genetic perspective, bidirectional Mendelian randomization analyses indicate that abdominal adiposity, rather than metabolic syndrome per se, is associated with intrapancreatic fat accumulation, supporting its role as an upstream determinant of pancreatic steatosis and the lipotoxic and inflammatory conditions that may facilitate KRAS-driven neoplastic progression [39].

### 4.4. Experimental Evidence from Preclinical Models

In a pancreas-specific transgenic mouse model, Peng et al. demonstrated that a sustained >2-fold increase in HuR expression is sufficient to induce early progressive fibro-inflammatory pancreatic remodeling, with chronic pancreatitis-like changes, acinar loss with fatty replacement, and age-dependent glucose intolerance, in the absence of PanIN lesions or pancreatic ductal adenocarcinoma (PDAC) even at advanced time points. Transcriptomic profiling revealed deregulation of more than 200 genes involved in inflammatory, immune, metabolic, and endocrine pathways, supporting a global reprogramming of the pancreatic microenvironment. Although HuR overexpression alone was not oncogenic, its combination with oncogenic Kras^G12D^ markedly accelerated neoplastic progression, resulting in a 3.4× increase in PDAC incidence compared with Kras^G12D^ alone, alongside earlier and more extensive PanIN development. Overall, these findings support a permissive, but not sufficient, role of HuR-driven inflammation and pancreatic fatty replacement in KRAS-dependent pancreatic carcinogenesis [23].

Consistently, other preclinical models have shown that loss of endogenous anti-adipogenic and anti-angiogenic regulators, such as pigment epithelium-derived factor (PEDF), in the setting of oncogenic KRAS promotes intrapancreatic steatosis, adipose-rich stromal remodeling, and progression to invasive pancreatic ductal adenocarcinoma, further supporting a causal role of metabolic-stromal dysregulation in lowering the barrier to malignant transformation [24].

### 4.5. Fibrotic Remodeling and Inflammatory Changes Associated with Pancreatic Steatosis

Translating these experimental observations to human disease, histological evidence summarized by Sreedhar et al. indicates that pancreatic ductal adenocarcinoma is frequently associated with marked fibrotic remodeling, reported in approximately 70–85% of specimens and significantly more prevalent than in non-cancer controls (85.5% vs. 41.8%, *p* < 0.0001). In contrast, inflammatory cell infiltration, while more common in cancer tissue overall, did not show a consistent association with intra-pancreatic fat deposition. These findings suggest that pancreatic steatosis is more closely linked to fibrotic remodeling of the pancreatic microenvironment than to inflammatory infiltration, although causality cannot be inferred [12,25].

## 5. Pancreatic Steatosis and Pancreatic Ductal Adenocarcinoma

### 5.1. Association Between Pancreatic Steatosis and PDAC

Available evidence consistently supports an association between intra-pancreatic fat deposition (IPFD) and pancreatic ductal adenocarcinoma (PDAC). Two systematic syntheses based predominantly on retrospective and cross-sectional studies first highlighted this relationship. In a systematic review of 13 retrospective studies including 2178 individuals, pancreatic fat deposition was detected in approximately 52% of patients with PDAC or premalignant lesions and was associated with a nearly threefold higher risk compared with non-cancer controls [12]. These findings were subsequently reinforced by a larger meta-analysis of 17 studies (2956 patients), which reported fatty pancreas in approximately 62% of pancreatic cancer cases and a more than 6-fold higher prevalence relative to non-cancer populations [13]. Although methodologically heterogeneous, these analyses consistently indicated a strong association between pancreatic steatosis and pancreatic cancer, while precluding conclusions on temporality and causality.

To address the lack of temporal ordering, Dong et al. conducted the first large population-based prospective analysis of IPFD and incident PDAC in the UK Biobank. In over 42,000 participants with baseline MRI assessment, higher IPFD preceded PDAC diagnosis and was independently associated with increased cancer risk. However, the association with PDAC was weaker than that observed for acute pancreatitis and diabetes mellitus and was limited by the small number of cancer events, relatively short follow-up, and reliance on a single baseline IPFD measurement, supporting temporal precedence but not definitive causal inference [8].

More recently, Yamazaki et al. extended this evidence by integrating a prospective MRI-based cohort study with Mendelian randomization analyses. Higher IPFD was associated with a substantially increased risk of incident PDAC independent of overall adiposity, and genetically determined increases in pancreatic fat were also linked to elevated PDAC risk. The convergence of prospective epidemiologic and genetic evidence lends support to a potential causal contribution of pancreatic steatosis to pancreatic carcinogenesis. However, the underlying biological mechanisms remain unclear, the proportion of IPFD variability explained by genetic instruments is modest, and residual pleiotropy cannot be entirely excluded, warranting cautious interpretation [9].

Beyond individual risk estimates, a recent systematic review of longitudinal cohort studies evaluated the population-level impact of excessive intrapancreatic fat deposition, specifically addressing temporal ordering between exposure and disease onset. Across 23 cohorts, excessive IPFD at baseline was consistently associated with an increased future risk of pancreatic cancer, with longitudinal studies reporting approximately a threefold higher risk compared with normal IPFD. Population-attributable fraction analyses suggested that nearly one-quarter of pancreatic cancer cases could theoretically be attributed to excessive IPFD, while E-value sensitivity analyses indicated that these associations were unlikely to be fully explained by unmeasured confounding. Nevertheless, heterogeneity in IPFD definitions, reliance on observational data, and the hypothetical nature of population-attributable fraction estimates require careful interpretation [14].

Complementing these population-based approaches, a recent US matched cohort study provided clinically intuitive evidence supporting a pancreas-specific risk profile associated with radiologically defined fatty pancreas disease. In this study, 82 patients with fatty pancreas disease were matched 1:2 with controls for age, sex, body mass index, and smoking status and followed for a median of 3.8 years. Despite comparable hepatic and subcutaneous fat content, intrapancreatic fat was markedly higher in the fatty pancreas group, and incident PDAC occurred exclusively among these patients (4.9% vs. 0%), with a median latency of approximately 2.7 years from index imaging. The absence of an excess of extra-pancreatic malignancies further supported an organ-specific association, although the small number of cancer events, retrospective design, and use of clinically indicated CT imaging precluded causal inference [15].

Beyond cancer incidence, emerging evidence suggests that intrapancreatic fat deposition may also be linked to metabolic and endocrine disorders, premalignant lesions and tumor aggressiveness.

### 5.2. IPFD and Metabolic and Endocrine Disorders

Type 2 diabetes mellitus is a recognized risk factor for pancreatic ductal adenocarcinoma and frequently coexists with intra-pancreatic fat deposition. In a large cross-sectional analysis from the UK Biobank, Gao et al. showed that pancreatic fat content was significantly higher in individuals with type 2 diabetes, but not in those with type 1 diabetes or pancreatic exocrine diseases, independent of demographic, lifestyle, and metabolic factors. Mediation analyses indicated that obesity and dyslipidemia only partially accounted for this association, supporting a close link between pancreatic steatosis and the diabetic metabolic context [26].

### 5.3. Pancreatic Steatosis and Premalignant Pancreatic Lesions

Evidence on premalignant pancreatic lesions is limited but suggests a consistent association with intra-pancreatic fat deposition. As summarized by Sreedhar et al., histological studies reported a markedly higher prevalence of intra-pancreatic fat deposition in pancreatic intraepithelial neoplasia (PanIN) compared with controls (71% vs. 13%, *p* < 0.0001). In intraductal papillary mucinous neoplasms (IPMNs), pancreatic steatosis has instead been evaluated indirectly using CT-based attenuation metrics. Furthermore, Kashiwagi et al. demonstrated significantly lower pancreatic attenuation values and pancreas-to-spleen ratios in IPMN patients than in individuals without cystic lesions, consistent with higher pancreatic fat content. Importantly, these findings derive from retrospective and cross-sectional data and therefore indicate an association rather than a temporal or causal relationship, leaving unresolved whether pancreatic fat accumulation precedes premalignant lesions, arises secondary to local pancreatic pathology, or reflects shared metabolic or inflammatory mechanisms [12,20].

### 5.4. Intra-Pancreatic Fat Deposition and Tumor Stage

Some evidence suggests that intra-pancreatic fat deposition is associated with a more advanced tumor stage in pancreatic cancer. Histological analyses reported a significantly higher adipocyte density in patients with nodal metastases compared with node-negative disease, while imaging-based studies showed lower CT attenuation of the pancreatic body and tail in N1 versus N0 patients, consistent with increased intrapancreatic fat content. Collectively, these findings indicate an association between pancreatic fat accumulation and nodal involvement. However, given the retrospective and cross-sectional nature of the available data, it remains unclear whether intra-pancreatic fat contributes to tumor progression or represents a secondary phenomenon related to locally advanced disease [12].

By contrast, in a recent hospital-based imaging study, pancreatic steatosis was identified in approximately 30% of patients with pancreatic ductal adenocarcinoma and was strongly associated with underlying chronic pancreatitis, but showed no independent association with tumor stage or overall survival. These findings argue against a direct role of intrapancreatic fat in determining tumor aggressiveness and instead suggest that pancreatic steatosis may reflect a pre-existing inflammatory or fibro-inflammatory substrate [40].

**Figure 2:**
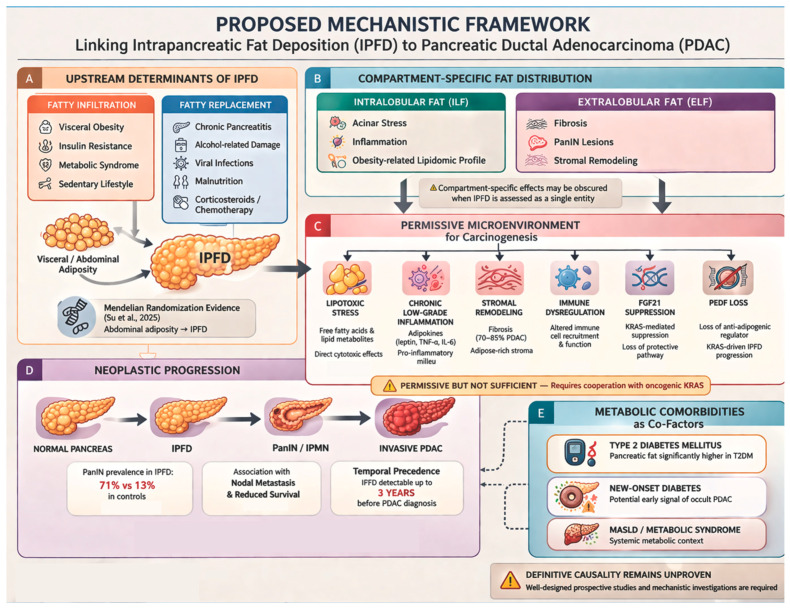
Proposed mechanistic framework linking intrapancreatic fat deposition (IPFD) to pancreatic ductal adenocarcinoma (PDAC). The figure integrates upstream determinants of IPFD (Panel **A**) [39], compartment-specific fat distribution (Panel **B**), the permissive microenvironment for carcinogenesis (Panel **C**), neoplastic progression (Panel **D**), and metabolic comorbidities as co-factors (Panel **E**). ELF, extralobular fat; FGF21, fibroblast growth factor 21; ILF, intralobular fat; IPFD, intrapancreatic fat deposition; IPMN, intraductal papillary mucinous neoplasm; MASLD, metabolic dysfunction-associated steatotic liver disease; PAF, population-attributable fraction; PanIN, pancreatic intraepithelial neoplasia; PDAC, pancreatic ductal adenocarcinoma; PEDF, pigment epithelium-derived factor; T2DM, type 2 diabetes mellitus.

The proposed mechanisms linking pancreatic steatosis and pancreatic cancer, derived from the more recent evidence, are summarized in Figure 2.

## 6. Imaging-Based Methods for the Quantification of IPFD

At present, no standardized imaging-based method exists for the quantification of intra-pancreatic fat deposition (IPFD). Nevertheless, advances in imaging techniques have enabled increasingly quantitative assessment of pancreatic fat, which would become particularly relevant for prevention and surveillance strategies should future studies confirm IPFD as a clinically meaningful risk factor [21,31].

### 6.1. Non-Contrast CT-Based Assessment of Pancreatic Fat

Using non-contrast computed tomography, intra-pancreatic fat deposition has been primarily assessed through the pancreas–spleen attenuation index (P.S100), with lower pancreatic attenuation reflecting higher fat content. In retrospective case–control studies, reduced pancreatic attenuation has been consistently associated with pancreatic ductal adenocarcinoma (PDAC), with risk increasing across higher degrees of fat infiltration and remaining independent of body mass index. Subsequent analyses extended these observations to the pre-diagnostic setting, demonstrating that pancreatic steatosis can be detected on CT scans up to several years before PDAC diagnosis, even after adjustment for metabolic confounders [6,16].

More recently, attenuation-based assessment has been refined through AI-assisted whole-gland pancreatic segmentation on non-contrast CT, enabling age- and sex-specific reference thresholds and overcoming the limitations of region-of-interest based approaches. Using this methodology, lower mean pancreatic attenuation was observed on pre-diagnostic CT scans of patients who later developed PDAC, independent of pancreatic volume, suggesting that increased intrapancreatic fat may precede overt tumor-related parenchymal changes [18].

The biological validity of attenuation-based CT metrics has been supported by studies correlating imaging findings with pancreatic histology. Fukuda et al. demonstrated that a low pancreas-to-spleen attenuation ratio accurately identified pathological fatty degeneration and was independently associated with PDAC risk, even after adjustment for metabolic confounders, supporting intrapancreatic fat as a genuine predisposing substrate rather than a tumor-related epiphenomenon [19].

Beyond invasive cancer, in a cross-sectional Japanese screening cohort, Kashiwagi et al. observed significantly lower pancreatic attenuation in subjects with intraductal papillary mucinous neoplasms compared with matched controls, independent of body mass index. Although attenuation was assessed locally rather than at the whole-gland level, these findings extend the association between intrapancreatic fat and pancreatic neoplasia to precursor lesions [20]. Taken together, these studies support the feasibility and reproducibility of non-contrast CT attenuation metrics for IPFD assessment, but conclusions are constrained by the retrospective nature of most studies.

### 6.2. MRI-Based Quantification of Pancreatic Fat

Quantitative MRI techniques enable direct, tissue-specific assessment of pancreatic fat content and have therefore been proposed as a more biologically informative alternative to attenuation-based CT metrics. Using proton density fat fraction (PDFF), Fukui et al. demonstrated a strong correlation between MRI-derived pancreatic fat estimates and histologically quantified intrapancreatic fat in non-tumoral tissue, with MRI outperforming CT attenuation in discriminating fat content. PDFF values were also higher in patients with pancreatic ductal adenocarcinoma than in controls and remained independently associated with cancer presence after multivariable adjustment, supporting the ability of MRI to capture clinically relevant pancreatic fat phenotypes [17].

Beyond cross-sectional validation, methodological studies have highlighted both the strengths and limitations of MRI-based fat quantification. Coe et al. showed that chemical shift-encoded MRI and proton magnetic resonance spectroscopy correlate with histological fat content, but also underscored substantial variability related to voxel placement, respiratory motion, and intrinsic pancreatic heterogeneity, indicating that reproducibility is highly protocol-dependent and requires careful standardization. Notably, pancreatic fat fraction showed only modest correlations with hepatic fat and anthropometric measures, suggesting that intrapancreatic fat is not simply a surrogate of systemic adiposity [41].

Importantly, MRI-based assessments have also been linked to pancreatic tissue remodeling, beyond bulk fat quantification. Kiemen et al. reported that MRI-estimated intrapancreatic fat correlates with acinar cell loss, increased collagen deposition, and a higher burden of precursor lesions such as PanIN and IPMN, suggesting that MRI-derived metrics may capture a broader fibro-fatty remodeling state of the pancreas [42].

Collectively, these findings position MRI as a powerful research tool for biologically contextualizing intrapancreatic fat deposition and its relationship to pancreatic remodeling and neoplastic risk. However, the lack of standardized acquisition protocols, limited whole-gland quantification, and scarcity of longitudinal data currently confine MRI-based pancreatic fat assessment to the investigational domain rather than routine clinical application.

### 6.3. EUS-Based Assessment of Pancreatic Steatosis

Endoscopic ultrasound (EUS) allows in vivo morphological assessment of pancreatic steatosis based on pancreatic echogenicity. Retrospective single-center studies using qualitative EUS criteria have reported a higher prevalence of fatty pancreas in patients with pancreatic ductal adenocarcinoma [43,44].

Some EUS-based analyses have further suggested that pancreatic echogenicity may vary across disease stages, with lower degrees of fatty infiltration observed in advanced cancer compared with early-stage disease, possibly reflecting progressive fibro-atrophic remodeling and cancer-related cachexia rather than a primary oncogenic effect [45].

However, the lack of standardized diagnostic thresholds, the operator-dependent nature of EUS assessment, and the restriction of available evidence to selected patients evaluated in tertiary referral settings substantially limit reproducibility and generalizability, and preclude reliable assessment of temporality or causality. Accordingly, the role of EUS in pancreatic cancer risk stratification or early detection remains undefined.

### 6.4. AI-Based Imaging Models for Quantification of IPFD

As previously noted, the absence of a standardized approach for IPFD quantification remains a key limitation. To address this issue, Joshi et al. systematically reviewed artificial intelligence-based imaging methods for intrapancreatic fat assessment. By comparing AI-derived measurements with manual reference standards across MRI- and CT-based studies, the authors showed that a considerable proportion of the variability and discordant findings in the existing literature stems from heterogeneous segmentation approaches, imaging protocols, and fat quantification metrics, rather than from true biological differences. In this context, AI-based automated IPFD measurement, although still technically suboptimal, has been proposed as a promising research tool with the potential to improve reproducibility and to enable large-scale and longitudinal studies, thereby supporting more robust investigations into pancreatic steatosis as a possible risk factor for pancreatic ductal adenocarcinoma [21].

## 7. Discussion

Despite the growing body of evidence linking intra-pancreatic fat deposition (IPFD) to pancreatic ductal adenocarcinoma, several critical limitations constrain interpretation and clinical translation. First, the literature is dominated by retrospective and cross-sectional studies, with only a limited number of prospective cohorts. While recent longitudinal and genetic studies support temporal precedence and suggest a possible causal role for IPFD, definitive causality cannot yet be established.

Second, substantial heterogeneity exists in the definition and assessment of pancreatic steatosis across studies, encompassing different imaging modalities, acquisition protocols, segmentation strategies, and thresholds. This methodological variability limits comparability between studies, contributes to inconsistent effect estimates, and complicates meta-analytic synthesis.

Third, residual confounding and reverse causation remain important concerns. IPFD frequently coexists with diabetes, subclinical pancreatitis, and early fibro-atrophic pancreatic remodeling, raising uncertainty as to whether pancreatic fat represents an upstream driver of carcinogenesis, a permissive substrate, or an early manifestation of occult disease.

From a biological perspective, although converging experimental data indicate that pancreatic steatosis interacts with oncogenic KRAS to lower the threshold for malignant transformation, the precise mechanistic pathways remain incompletely defined [46].

It is still unclear which components of pancreatic steatosis—adipocyte infiltration, lipotoxic stress, inflammatory signaling, or fibro-fatty remodeling—are necessary or sufficient to promote neoplastic progression in humans.

Finally, despite accumulating evidence suggesting that IPFD may represent an independent risk factor for PDAC, the absence of validated risk prediction models integrating pancreatic fat with clinical, metabolic, and genetic factors precludes its use for targeted screening or surveillance. Until causality, biological specificity, and predictive performance are firmly established, IPFD should be regarded as a promising but unvalidated marker within the broader challenge of identifying pancreatic cancer at a curable stage.

## 8. Conclusions and Future Directions

Taken together, current evidence consistently links intrapancreatic fat deposition to pancreatic ductal adenocarcinoma risk, supporting a strong suspicion that pancreatic steatosis may represent an independent risk factor for PDAC; however, this has not yet been definitively established for the reasons outlined in the limitations discussed above.

Future research should prioritize robust prospective studies designed to establish causality, strengthen temporal ordering, and define how early pancreatic steatosis emerges relative to PDAC development. In parallel, standardized and reproducible methods for IPFD quantification are needed to enable reliable risk stratification across populations and imaging modalities. Finally, dedicated mechanistic studies are required to elucidate the biological pathways linking pancreatic steatosis to pancreatic carcinogenesis and to identify potential therapeutic targets.

Addressing these gaps could enable two major advances in the PDAC field: the development of risk-based screening and surveillance strategies for individuals with IPFD and the exploration of preventive or therapeutic interventions targeting pancreatic steatosis itself.

## Figures and Tables

**Table 1:** Main studies and their key findings.

Study	Sample Size	Key Findings
**Systematic reviews and meta-analyses**		
Sreedhar, 2020 [12]	13 studies, 2178 individuals	IPFD present in ~52% of PDAC/premalignant cases; ~3-fold higher risk vs. controls
Lipp, 2023 [13]	17 studies, 2956 patients	FP prevalence in PC: 62%; >6-fold higher prevalence vs. controls; reverse association (PC risk in FP patients) not significant
Leybourne, 2025 [14]	23 cohorts, >100,000 participants	3-fold higher PDAC risk with excessive IPFD; ~23% of PDAC cases potentially attributable to excessive IPFD
**Prospective cohort studies**		
Dong, 2024 [8]	42,599 participants	Elevated IPFD independently associated with increased PDAC risk; FP associated with ~2-fold higher PDAC risk
Yamazaki, 2024 [9]	MRI cohort + genetic analysis	Higher IPFD associated with PDAC risk independent of overall adiposity; genetically determined IPFD also linked to PDAC
Chatterjee, 2025 [15]	82 FPD patients, 164 controls	PDAC occurred exclusively in FPD group; no excess extra-pancreatic malignancies, supporting organ-specific risk
**Case-control and cross-sectional studies**		
Hoogenboom, 2021 [16]	32 PDAC cases, 117 controls	Pancreatic steatosis detectable on CT up to 3 years before PDAC diagnosis; ~3-fold higher risk vs. controls
Desai, 2020 [6]	PDAC patients and controls	Lower pancreatic attenuation (higher fat) associated with PDAC; risk increased with higher fat infiltration, independent of BMI
Mathur, 2009 [7]	Resected PDAC specimens	Higher intrapancreatic adipocyte density associated with nodal metastasis and worse survival, independent of BMI
**Imaging-based quantification studies**		
Fukui, 2019 [17]	55 patients	MRI-PDFF strongly correlated with histological fat and outperformed CT attenuation; PDAC patients had higher pancreatic
Janssens, 2021 [18]	469 CT scans	Pre-diagnostic PDAC scans showed lower pancreatic attenuation; AI-assisted whole-gland segmentation enabled age/sex-specific thresholds
Fukuda, 2017 [19]	PDAC patients and controls	Low pancreas-to-spleen attenuation ratio correlated with pathological fatty degeneration and independently predicted PDAC risk
Kashiwagi, 2018 [20]	IPMN patients vs. controls	Lower pancreatic attenuation in IPMN patients vs. controls, independent of BMI
Joshi, 2025 [21]	12 studies, >50,000 participants	Variability in IPFD literature largely stems from heterogeneous protocols rather than true biological differences; AI promising for standardization
**Mechanistic and pathophysiological studies**		
Frendi, 2024 [22]	Human pancreatic tissue	Intralobular fat linked to acinar stress/inflammation; extralobular fat correlated with PanIN burden and fibrosis
Peng, 2018 [23]	Mouse model	HuR overexpression induced fibro-inflammatory remodeling and fatty replacement; combination with KRAS increased PDAC incidence 3.4-fold
Grippo, 2012 [24]	Mouse model	Loss of PEDF with oncogenic KRAS promoted IPFD, adipose-rich stromal remodeling, and progression to invasive PDAC
Tomita, 2014 [25]	Resected pancreatic specimens	Fatty degeneration and fibrosis significantly more prevalent in PDAC than in non-cancer controls
**Metabolic and endocrine associations**		
Gao, 2025 [26]	61,088 participants	Pancreatic fat significantly higher in T2DM but not T1DM; obesity and dyslipidemia only partially explain the association

## Data Availability

The original contributions presented in this review are included in the articles. Further inquiries can be directed to the corresponding author(s).

## Abbreviations

The following abbreviations are used in this manuscript:

PDAC	Pancreatic ductal adenocarcinoma
ILF	Intra-lobular fat
ELF	Extra-lobular fat
PanIN	Pancreatic Intraepithelial Neoplasia
IPFD	Intra-pancreatic fat deposition
IPMN	Intraductal papillary mucinous neoplasm
PDFF	Proton density fat fraction
